# Identifying Criteria for the Evaluation of the Implications of Brain Reading for Mental Privacy

**DOI:** 10.1007/s11948-017-0003-3

**Published:** 2017-12-15

**Authors:** Giulio Mecacci, Pim Haselager

**Affiliations:** Donders Institute for Brain, Cognition and Behaviour, Montessorilaan 3, 6525 HR Nijmegen, The Netherlands

**Keywords:** Brain reading, Neuroethics, Criteria, Mental privacy, Societal implications, Neuroimaging

## Abstract

Contemporary brain reading technologies promise to provide the possibility to decode and interpret mental states and processes. Brain reading could have numerous societally relevant implications. In particular, the private character of mind might be affected, generating ethical and legal concerns. This paper aims at equipping ethicists and policy makers with conceptual tools to support an evaluation of the potential applicability and the implications of current and near future brain reading technology. We start with clarifying the concepts of mind reading and brain reading, and the different kinds of mental states that could in principle be read. Subsequently, we devise an evaluative framework that is composed of five criteria-accuracy, reliability, informativity, concealability and enforceability-aimed at enabling a clearer estimation of the degree to which brain reading might be realistically deployed in contexts where mental privacy could be at stake. While accuracy and reliability capture how well a certain method can access mental content, informativity indicates the relevance the obtainable data have for practical purposes. Concealability and enforceability are particularly important for the evaluation of concerns about potential violations of mental privacy and civil rights. The former concerns the degree with which a brain reading method can be concealed from an individual’s perception or awareness. The latter regards the extent to which a method can be used against somebody’s will. With the help of these criteria, stakeholders can orient themselves in the rapidly developing field of brain reading.

## Introduction

Mind reading is as old as social interaction. In daily life, we are constantly trying to understand the beliefs, desires, intentions, feelings and capacities of other agents (either human or animal). Traditionally, informal observation of an agent’s behavior (including language production) provided the sole basis for the ascription of mental states. In the last century, such an informal approach has been gradually complemented, and sometimes replaced, by systematic psychological observation and testing. These methods were introduced and commonly employed to assess an individual’s mental health, or potential for education and career (Gross 1962). Over the last decades, brain measurements have become a further source of information. They can be used to diagnose pathologies, develop cognitive theories, drive software or hardware devices, or infer the occurrence and nature of certain mental states. The act of making inferences regarding the occurrence and nature of mental states has recently been referred to as ‘brain reading’ (Haynes 2012): the observation of brain structure and/or activity aimed at obtaining insights about mental states.^1^ In the remainder of this paper, we will speak of ‘brain reading’ to refer to the use of brain measurements for the purpose of mind reading, in distinction from other forms of mind reading (such as behavioral observation). So, whereas mind reading is the attempt to understand mental states, brain reading is the attempt to mind read solely on the basis of brain measurements. This additional possibility might have significant implications for the private character of the mind, at least in principle challenging the widely shared intuition that our mental states can be secluded. Ayer (1963, chapter 3) distinguished at least four ways in which our mental states can be said to be private.^2^ First, they are private in the sense that they can be *incommunicable*. People can experience insurmountable difficulties in adequately expressing their thoughts or feelings. There is, or there can be, a felt difference between the report and the experience of what is reported. Second, mental states are private in the sense that individuals have a ‘first person perspective’ (Shoemaker 1988, 1994) on their inner mental life. Each person only has such ‘*special access*’ to his or her own mental states. One knows introspectively about one’s own mental states, which is different from the way anyone else can know about them. In other words, there is a qualitative component that is inaccessible to an external viewer. Third, mental states are private in the sense that they can be *unshareable*, meaning that it is impossible for two persons to entertain exactly the same thought in exactly the same way. Fourth, mental states are private in the sense they can be *incorrigible*, for certain knowledge claims cannot be corrected or overridden. There seems to be no way to categorically deny subjective reports of thoughts and sensations. “That’s how I feel it” is a statement that, in many cases, invokes an unassailable authority regarding one’s own mental life.

Brain reading’s potential implications for mental privacy have recently captured the interest of both the popular (Roth 2009; Sample 2012; Wolpe 2009) and the scientific press (Farah et al. 2009; Haynes 2012; Shen 2013), generating hype and expectations (hopes as well as fears) in society. On the one hand, brain reading technology might lead to a number of clinical and scientific advances regarding the nature of mental states and their neural representations.

On the other hand, however, it could generate a number of ethical concerns, from the potential use and abuse of collected personal data (Ienca and Haselager 2016) to Orwellian scenarios where peoples’ liberties are at stake and minds can be coercively or covertly monitored (Federspiel 2007). For instance, as Shen reports, one of the questions that is commonly discussed in newspapers and mass media is whether ‘brain science [will] be used by the government to access the most private of spaces—our minds—against our wills’ (Shen 2013, p. 654). It is not difficult to imagine how the possibility to extract thoughts from the brain without appealing to behavioral cues can be unsettling. Mental privacy infringement has been discussed earlier in relation to psychological profiling and polygraph testing (Black 1994; Hermann 1971). Those techniques aim at mind reading without directly accessing brain functioning. Contemporary technologies that monitor brain activity constitute a significant addition to those indirect techniques in that they can establish an explicit relationship between psychological processes and the underlying neural events. If one accepts the assumption that every mental state must be implemented by some neural mechanism, then the observation of the causal machinery underlying thought and feeling may be considered by some to be a good or even compelling reason to override the traditionally decisive first-person reports. This could be further strengthened by the potentially superior performance of brain reading methods over classic investigation techniques. In addition, direct observation of the brain could lead, at least in principle, to access a significantly vaster or more detailed array of mental states.

However, it is far from clear that the unsettling consequences sketched in the media may actually take place within a reasonable time frame. The technical and theoretical challenges for brain reading are considerable. Indeed, it is not difficult to find contrasting claims regarding the achievements of brain reading within the same paper (Haynes 2012). That said, it is also important to discuss potential implications of a technology that is still in its infancy, if only to avoid committing a—well-known to the ethics of technology—delay fallacy (van de Poel and Royakkers 2011). Ideally, discussions about the potential implications of brain reading should take place in parallel with, and not after, the development of the technology. Therefore, this paper aims at equipping ethicists and policy makers with conceptual tools to support an evaluation of the potential applicability and the implications of current (as of 2018) and near future (approximately 5–10 years, based on currently ongoing research) brain reading technology. We devise an evaluative framework that is composed of five criteria—accuracy, reliability, informativity, concealability and enforceability—aimed at enabling a clearer estimation of the degree to which a certain technology could be applied in contexts where mental privacy could be at stake. In particular, our conceptual framework aims at facilitating judgments on: (1) the applications where brain-reading could be more reliably used (e.g. which states of mind can be more easily investigated); (2) the different contexts where it could be applied (e.g. in court cases or for job assessments); (3) the possible time frame within which different applications could be expected (focusing on now or in the near future); (4) the degree to which these applications could pose a threat to mental privacy. The five criteria of the evaluative framework we propose can be effectively employed by stakeholders such as ethicists, legal experts and policy makers, who require instruments to aid practical ethical reasoning and decision making, or to systematize and clarify their questions regarding a developing neurotechnology. A proper understanding of brain reading methods, their potential and their limits, maximizes the chances to timely assess and address emerging ethical, legal and societal implications.

## Brain Measurements, Mind Reading and Brain Reading

In this section, we aim to explain some of the basics about brain reading methods and technologies^3^ and provide a clear terminology to talk about them. In order to understand what brain reading is, we should start from the more general category of ‘brain measuring’.^4^ With that term, we describe any process or technology aimed at obtaining information about the brain and/or its functioning, from direct observation (e.g. autopsies or exploratory surgery), to modern imaging technologies (e.g. magnetic resonance imaging, positron emission tomography…). Brain measurements are useful in at least four domains of application. The first one is the production of physiological and pathological models and theories. This is of importance to address clinical cases and advance medical knowledge. A second domain where brain measurements can play a role is the development of cognitive theories. Cognition could in principle be studied independently from the specific structures that realize it, be it a brain, a chip, or whatnot. However, observing how the brain works can contribute to inform and/or constrain cognitive theories. Information about the brain can provide inspiration for producing biologically plausible cognitive models. A third domain where brain measurements are central is Brain–Computer Interfacing. Brain–Computer Interfaces (BCIs) are technologies aimed at “utilizing” brain signals to command a software or hardware device (e.g. to control the movement of a cursor on a computer screen). Additionally, those brain signals can be used to provide “neurofeedback”. A user can visualize her own ongoing brain activity in order to learn self-regulation of brain functions or be warned about imminent undesired neural events, e.g. epileptic seizures. In BCI a machine learning algorithm recognizes and categorizes, an arbitrary, preferably easy to evoke and measure, neural activity pattern. The particular kind or nature of the mental state that is correlated to such activity need not be relevant as long as it can be used to reliably drive a system^5^ or provide a user with a certain feedback. Finally, brain measurements can be used as brain reading: a brain measurement aimed at mind reading. Brain reading, though sharing numerous techniques with BCI, differs from it in its main scope. Rather than utilizing brain markers as inputs for a device, brain reading aims at understanding the way mental states are represented in the brain. That is, brain measurements are used to *decode or interpret* mental states (assess their nature and/or content). As suggested by the very word ‘reading’, brain reading is based on interpreting (combinations of) neuronal signs and drawing inferences about their meaning. As indicated, traditional mind reading activity relies on behavioral observation and inferential processes. Brain reading technology allows one to replace the observation of behavior with measurements of the brain structure and/or activity. Assuming a correlation between brain structure and functions and mental states, the latter can be inferred from the observation of the former.

**Fig. 1:**
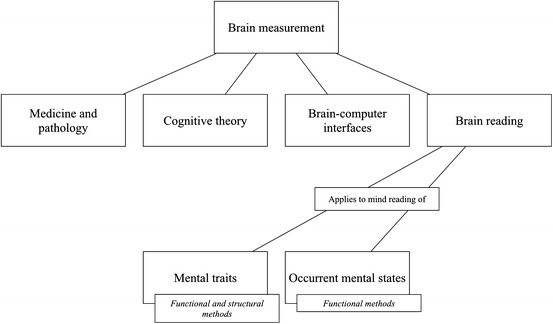
Different applications of brain measurement

In this paper, we use the concept of ‘mental state’ in a rather broad fashion to encompass every aspect of an individual’s psychology, including, but not limited to, personality traits and dispositions (e.g. sexual preferences, personal tastes and habits…), qualitative states (e.g. perceptions, emotions, feelings…), propositional states (e.g. knowledge, beliefs), intentions and goals, plans, memories etc. However, we should keep an important distinction in mind. On the one hand, we have *traits* that are relatively permanent dispositional psychological qualities, characteristics of individuals. On the other hand, we have *occurrent* mental *states*, the states that are entertained or experienced by a subject at a particular moment in time. Psychological traits enable and dispose a subject towards entertaining a certain occurrent mental state. For instance, having a high degree of trait anxiety makes a subject more vulnerable to experiencing anxiety in a variety of contexts and situations. Traits are also conceivable as capacities or enabling conditions. For instance, having a certain degree of self-control is a trait that makes a subject capable of displaying self-control in certain situations where other subjects might be unable to. Below, we will talk about mental states in general to refer to both traits and occurrent states, unless otherwise specified (Fig. 1).

A further important distinction is that between apparent and non-apparent mental states: apparent mental states are those that can be identified by mere external observation of behavior, thanks to normal human mind reading capacities, e.g. by seeing trembling hands inferring that someone is nervous. Non-apparent mental states, instead, are concealed from external observation. Furthermore, different technologies have been developed to identify both apparent and non-apparent mental states without appealing to *human* mind reading capacities. Identification of apparent mental states can for instance be automatized by using deep learning techniques for feature recognition (Güçlütürk et al. 2016), achieving performances that can get close to, or become better than, human ones. Applied to brain reading, such methods offer, at least in principle, an additional window on apparent mental states, potentially more penetrating than introspection or external observation. Brain reading methods can also offer distinctive insight into non-apparent mental states and this could make those methods particularly relevant from e.g. a clinical perspective [e.g. assisting psychotherapy by providing a better understanding of a patient’s psychological complexities (Habes et al. 2013)], but also more compelling from an ethical, legal and societal perspective. Existing methods, such as the polygraph (Vicianova 2015), infrared thermal imaging (Park et al. 2013) and voice stress analysis (Ruiz et al. 1990), sport principled limits when compared to brain reading technology. They typically only allow the detection of a generic physiological alteration, commonly associated—to different extents—with particularly compelling emotional states. Technologies that directly record neural dynamics could provide significantly more detailed information about one’s apparent and non-apparent states of mind.

Different mental states can be assessed with different brain reading methods. In assessing traits, brain measurements can reveal to a certain extent the presence -or the probability thereof- of certain features that characterize or underlie the attributes or behavioral dispositions of a person (Ma et al. 2014), such as intelligence (Malpas et al. 2016), self-control (Krämer and Gruber 2015; Maier et al. 2015), sexual orientation (Habermeyer et al. 2013; Poeppl et al. 2015; Ponseti 2012) etc. This can be done either by checking for certain brain anatomical features (through either neurobiological analysis or by the means of structural imaging technologies), or by identifying characteristic patterns of neural activation that can be associated to certain traits. An organic anomaly, a tumor, a stroke, or just a particular feature of the cerebral tissue (such as e.g. the amount of white matter in a particular neural pathway), might contribute to inferring an individual’s (in)capacity to display a certain behavior, e.g. to maintain a conduct that is considered to be normal. For instance, Motzkin et al. (2011) suggest that “psychopathy is associated with reduced structural integrity in the right uncinated fasciculus”. In healthy brains, structural and neurobiological markers such as density or connectivity of the neural tissue in certain areas, could be found to predict the presence of certain cognitive traits (Bernardi et al. 2014) [similar processes have been famously applied in genetics (Farahany 2016; Illes and Racine 2005; Rigoni et al. 2010)]. Whereas traits can be in principle found out to be connected to both structural or functional neural features, occurrent mental states are always connected to brain activity itself. Those states are defined as temporary functional states of the brain, and are often identified through functional neuroimaging technology that allows one to monitor, classify and interpret the neural activity. So far, different functional methods have been applied to the investigation of numerous mental states such as, but not limited to, intentions (Bode et al. 2012, 2013; Haynes et al. 2007; Soon et al. 2008, 2013), visual perceptions (Kay et al. 2008; Nishimoto et al. 2011; Schoenmakers et al. 2013), memories (Peth et al. 2015), active semantic knowledge (Carlson et al. 2014; Huth et al. 2012, 2016; Mitchell et al. 2008), emotions (Huis in ‘t Veld and de Gelder 2015; Plassmann et al. 2008), political preferences (Lamme 2010), dreams (Horikawa et al. 2013), pain (Cowen et al. 2015; Reardon 2015; Salmanowitz 2015; Wager et al. 2013), and (levels of) consciousness (Blume et al. 2015).

The general approach could be simplified as follows (see also Poldrack and Farah 2015). A brain monitoring device (e.g. electroencephalography [EEG], functional magnetic resonance imaging [fMRI]…) is used to collect information about a person’s neural activity that co-occurs with the expression of (a) certain mental state(s). Once the data have been recorded, different statistical methods and machine learning techniques allow one to analyze the collected information and create a representational map that connects the targeted mental states to the concurrent neural events. The mapped associations between the targeted mental states and the neural activity are learned and generalized to a certain extent. Once this procedure is complete and successful, one can apply the trained algorithm to categorize and decode mental states on the basis of the observation of the neural activity measurements.

## Assessing the Implications of Brain Reading Applications for Mental Privacy

Having a clearer idea of what brain reading can and cannot do, it is important to form an opinion on whether, when and to what extent it could be applied, both now and in the near future. Also, a clearer picture would help determining at what point specific concerns may become relevant or urgent. Whether or not a certain technology might be adopted for certain applications, depends on numerous contingent factors, and drawing a complete model would be beyond the scope of this paper. However, we isolate five important factors that might influence brain reading’s practical adoption, especially in those contexts where mental privacy is at stake: accuracy, reliability, informativity, concealability and enforceability. Unlike factors like price, availability, hype etc. (on which we will not focus in this paper), these five factors are bound to technical and theoretical limits and possibilities. Ideally, they are meant to constitute a framework to aid non-experts in asking relevant and meaningful questions. They can potentially assist stakeholders with different backgrounds in understanding future advances of brain reading, and in evaluating how realistic certain practical applications might be. For policy makers in particular, these five principles could aid the production of scientifically informed guidelines.

In order to be considered for adoption in those contexts where possible implications for mental privacy might occur, brain reading methods must achieve certain performance standards. Different contexts of application will have different requirements. Evaluating the performance of a certain technology can become a rather technical question, and moreover one that can become quickly outdated because of ongoing research and development. The notion of performance is well known to experts in neuroimaging, and is commonly divided into two components, *accuracy* and *reliability*. We propose to add a third one, *informativity.* While accuracy and reliability have to do with how well a certain method can read what is meant to be read, informativity concerns the relevance the obtainable data has for the practical purposes for which the method is meant to be used. Two more criteria, that specifically make sense in relation to those scenarios where the main preoccupation could be the violation of mental privacy and civil rights, are *concealability* and *enforceability*. While the former concerns the degree with which a certain brain reading method can be concealed from an individual’s perception or awareness, the latter regards the extent to which a method can be used against somebody’s will (Table 1).

**Table 1:** Criteria for the assessment of the implications of brain reading for privacy

	Type of datum	Indicates	Aids decision making by
Accuracy	Quantitative	The percentage of times a certain brain reading method correctly identifies a state of mind	Contributing to an assessment of how trustworthy a result of a brain reading is. This, in turn, helps understanding the extent to which decisions about individuals may or may not be based upon their brain readings
Reliability	Quantitative	The extent to which the method’s results remain unaltered over time and across different subjects	Contributing to an assessment of how the validity of a brain reading method remains trustworthy while applied to a larger spectrum of subjects. This, in turn, would be informative on how generalizable the application is
Informativity	Qualitative	The relevance of produced information for the purposes at hand	Contributing to an assessment of whether and to what extent a brain reading method can answer specific questions. This, in turn, is informative on whether particular brain reading methods might be suitable for specific applications
Concealability	Qualitative	The extent to which a brain reading method could be used unbeknownst to a subject	Contributing to an assessment of whether and to what extent a method is applicable in scenarios where there is an interest in covertly extracting information from a subject. This is particularly relevant in the discussion on mental privacy and personal data security
Enforceability	Qualitative	The extent to which a brain reading method could be used against somebody’s will	Contributing to an assessment of whether and to what extent a method is applicable in scenarios where there is an interest in extracting information from a subject coercively. This is particularly relevant in the discussion on mental privacy and personal data security

### Accuracy, Reliability, Informativity

*Accuracy* can be represented as the percentage of times a certain method correctly identifies states of mind that are targeted for decoding.^6^ Here, it is important to consider that in certain cases the specific *types of error* reducing the accuracy matter a great deal. In particular, the relative presence of false positives and false negatives can be of great importance in relation to certain contexts of application, e.g. in law. Whereas a court might be inclined to risk by admitting a method that can lead to a small percentage of false negatives, it might be less willing to base a decision on or even just consider a method that is prone to false positives, which could imply convicting an innocent person.

The concept of *reliability* expresses the extent to which the method’s results (e.g. identification of a particular trait) remain unaltered over time and across different subjects. Achieving high reliability usually represents a challenge, given the plasticity of individual brains and the large differences that can exist between different subjects.

When considering performance in the context of practical applicability, accuracy and reliability are not the only important criteria. It is also essential to capture and express how relevant the outcomes of a certain technology or method can be for a specific application. This question regards the amount and nature of the information that can be obtained through brain reading, rather than its correctness. We express this with the concept of *informativity*. Informativity regards the amount of produced information relevant for the purposes at hand. Whereas accuracy can be expressed quantitatively, informativity is a qualitative measure that depends on the question one wants to answer. Factors like number, kind and level of detail with which mental states can be identified, are all important in evaluating how informative, and hence potentially applicable, a certain method can be for a given scope. For instance, Huth et al. investigated how the meaning of language is represented in the brain. The outcomes of this study suggest that “the contents of thought, or internal speech, might be decoded” (Huth et al. 2016). Huth et al. indicate that different cortical areas encode with variable accuracy for semantic mental states belonging to 12 semantic domains, such as ‘person’, ‘visual’ or ‘time’. Their results constitute a remarkable advance in neuroscientific research. However, the degree of abstractness and the number of states they were able to discriminate would be a major discussion point in considering current practical applicability. Although this method could be used to gain some insight in whether a subject is at a certain moment thinking of one of the 12 categories (e.g. a person or a car), it is currently unclear whether one could be able to determine *which* car or *which* person. Those details may indeed be represented in the brain by different patterns of activity, but with the current method, cutting edge as it is, this cannot be discriminated.

The three aforementioned criteria constitute a coherent set that is worth discussing in more detail before continuing with the other two criteria. As mentioned at the beginning of this section, evaluating the performance is only possible in relation to a given particular context of application. Different contexts might have different requirements in regard to their level of trustworthiness, type and quantity of the provided information. In contexts where the stakes are extremely high, such as criminal law, high levels of performance will be required before its results can be taken into account. There, the methods applied in brain reading technology, in order for its results to be admitted (as regulated by e.g. the Daubert standard (*Daubert v. Merrell Dow Pharmaceuticals (92*-*102), 509 U.S. 579* (1993)) is required to be reliable (in our terminology, accurate and reliable) and relevant (our ‘informativity’). Moreover, the practical application of brain reading should be generally accepted by the scientific community. As of yet, one may have substantial reasons to doubt that such is the case.

We want to discuss a practical scenario that considers a brain reading method that promises the challenging and societally relevant capacity to identify subjects with pedophilia. That particular sexual preference, for its social significance and its potential legal consequences, can be, and normally is, concealed by the subjects entertaining it. In a recent experiment, Ponseti et al.’s (2012) used fMRI to identify admitting^7^ pedophiles by detecting states of sexual arousal in brain activity. They monitored the subjects’ reactions to the presentation of pictures of potential sexual partners of different gender and age. In terms of accuracy, 95% of the subjects were correctly classified, and the classification produced no false positives. The performance remained solid across the subjects included in the investigation, although the reliability over time within a subject has to our knowledge not yet been assessed. The relatively high performance may be due to the fact that this particular method, rather than identifying the neural correlates of the different sexual preferences, indirectly infers sexual preferences by discriminating a general state of arousal against a baseline. It is on the nature of the presented pictures, the evoking stimuli, that the inference about a certain sexual preference is drawn. The level of detail and the amount of relevant information obtainable in this way is such that some practical applications are foreseeable. For instance, it would be a useful tool in therapeutic or rehabilitative contexts, e.g. to monitor patients’ or convicts’ response to therapy. However, in other contexts, such as e.g. criminal law, this method’s performance might still be considered insufficient.

To our knowledge, this brain reading based method to assess pedophilic tendencies has never been proposed in court, but considering the law’s reflection on an older technology provides some insights. The one century old penile plethysmography (or phallometry) is a technology that aims at detecting states of sexual arousal. Rather than directly measuring neural events, it detects variations in penis’ diameter or volume. It is known to have average accuracy comparable to that of Ponseti et al’s method.^8^ Yet, phallometry has been previously deemed unacceptable as evidence in the American Common Law [see e.g. U.S. v. Powers (*United States v. Powers, 59 F.3d 1460* (*4th Cir.*^1995^), n.d.)].^9^ The fact that the performance of the two methods is similar, both in the nature of the information provided and in the overall accuracy, may be taken to suggest that the brain reading based method will not be accepted in court as well, based on purely performance based criteria.^10^ In addition, the two methods have been claimed to show different proneness to false positives and false negatives. In the discussion of their paper, Ponseti et al. maintain that their method produces no false positives while phallometry produces no false negatives [although it must be noticed that the accuracy of phallometric assessments is evaluated differently in different papers (Ponseti 2012)]. A further detail that has to be considered is that Ponseti et al.’s performance is achieved with fully admitting, fully cooperative subjects. As we will further discuss in the next section, with individuals that try to actively conceal their sexual preference, emotional and physiological reactions, the performance would likely decrease to a significant extent (noticeably, this is also the case for classic phallometric assessments).

Assessing the accuracy, reliability and informativity of a brain reading method in order to decide on the practical usability of its results (e.g. in legal cases) is a multi-faceted task that cannot be answered once and for all, but requires highly context specific considerations. We now return to the remaining criteria, concealability and enforceability, that didn’t need to be taken into account in the legal scenario we analyzed above. However, the possibility to apply brain reading methods without or even against somebody’s consent is an important part in understanding and evaluating their practical applicability in those scenarios where mental privacy and civil rights are more at stake.

### Concealability and Enforceability in Non-cooperative Scenarios

The possibility of collecting personal data secretly or against somebody’s will is not a novel concern, and it has been discussed since the end of the nineteenth century. Warren and Brandeis, as early as 1890, were denouncing how “instantaneous photographs and newspaper enterprise […] invaded the sacred precincts of private and domestic life; and numerous mechanical devices threaten[ed] to make good the prediction that “what is whispered in the closet shall be proclaimed from the house-tops.”” (Warren and Brandeis 1890). In essence, their concern focused on the possibility that someone’s private matters unwillingly could become public due to new technological possibilities. It is worth reflecting on the extent to which brain reading could be used unbeknownst to a subject (a criterion that we will call *concealability*) or against somebody’s will (its *enforceability*). These two aspects are especially relevant when considering potential abuses of the technology and the relative preventive strategies.

Brain reading technology might to a certain extent be used without an individual being aware of it, or even being aware that any brain measurement is taken at all. Different types of scenarios can be devised. In the most innocent, and perhaps most currently common one, personal information can be incidentally discovered in the course of conducting scientific research or medical interventions with completely aware and cooperative subjects. Incidental findings fall by definition beyond the scope of an intended application. Usually, these findings consist of previously undiagnosed neural pathologies, but can in principle be regarded as aspects of one’s psychology as well. The extent to which the information obtained is passed to—or retained from—the subject is currently regulated in different protocols and ethical guidelines of neuroimaging (Bos et al. 2016; Illes 2006; Shoemaker et al. 2011). Findings need not to be accidental, for a malicious user could intentionally collect data that fall outside the scope of a particular application for which the subject has given expressed consent. This does not necessarily apply only to scientific studies but could also involve more common scenarios. One example concerns consumer-grade brain computer interfaces (Ienca et al. 2017). Small and relatively cheap EEG devices started circulating among the public at large a few years ago (Emotiv 2017; Neurosky 2017), marketed as hands-free controllers for gaming and computer applications. Similar consumer-grade appliances already have the potential to be utilized to collect personal data without the user’s consent and awareness (Martinovic et al. 2012). For completeness sake, additional scenarios are possible, where ill-intentioned scientists use brain measurements of e.g. unconscious or partially conscious subjects without their consent, or use yet to be developed technologies enabling brain measurements of awake free moving subjects without being detected. As indicated earlier, we restrict ourselves to more near-future brain reading applications and therefore refrain from discussing these more futuristic scenarios.

The above listed scenarios are sorted by their decreasing level of cooperativity from a subject. While in accidental findings a subject is actively following the experimenters’ requests, awake and unknowing subjects behave normally and freely engage in a number of daily cognitive tasks. It is important to notice that the less a subject is actively cooperating in performing a certain mental task, the harder it generally becomes to collect meaningful data. Non-cooperation would be likely to lead to a significant decrease in performance, due to the general difficulty of discriminating neural process in a subject that is not actively focusing on a single task.

A further step towards non-cooperation involves cases where subjects are well aware that a reading is happening, but actively try to defy the technology. *Enforcing* brain reading is not easy, as brain reading methods, and particularly those that are based on functional assessments (e.g. fMRI), are prone to different kinds of intentional disruption. The simplest way one could render results of a functional imaging method invalid would be by generating noise. Noise can be generated for instance by simple muscular movements. A sufficient level of noise would likely make the entire dataset unusable. Normally, this form of ‘sabotage’ is relatively easy to discover, when one participants’ dataset contains significantly more noise than the datasets of other participants (unless all the subjects aim to sabotage the brain reading process). One could also deliberately refrain from producing the investigated brain signals, by not performing the cognitive task accurately, or only some of the time, or by focusing on other cognitive tasks, engaging in mind wandering, etc. Even simple shifts in attention have been shown to warp the way mental states are represented in the brain (Çukur et al. 2013). Here, discovering the lack of cooperation may still be possible, but more difficult than in the case of deliberate noise production.^11^ Brain reading procedures can not only be sabotaged by a subject that is forced to undergo it. It is during research and development that a technology becomes particularly vulnerable to being misled or ‘boycotted’. The data collection phase is particularly sensitive in that regard: one or more research subjects might declare full cooperation while covertly devising the process by deploying one of the mentioned sabotage strategies. This could be due for instance to privacy concerns and result from some kind of political activism.^12^ At this particular stage, for many mental states there is no other way to know which ones are entertained by a subject but to ask for subjective reports and assume complete cooperation. When one is to map mental states to neural activity, any uncertainty about the former drastically reduces the chances of success. At the research stage, the performance of the method itself is assessed, and hence depends on, the cooperation and *bona fide* of the experimental subjects. The process can be boycotted at different stages of the research phase itself, as any method commonly undergoes a number of validations and tests, all based on subjective reports.

As mentioned while discussing Ponseti et al. and their study on sexual preferences, research professionals are well aware of the issues regarding different non-cooperative scenarios. Further research is needed to assess the extent to which the performance of brain reading methods can be preserved as subjects actively try to defy them. Advances in neuroscience and technology might partially address those technical limitations. Neuroimaging research is for instance increasingly recognizing the value of methods aimed at decoding mental states under more natural, ecological conditions (Nishimoto et al. 2011; Stansbury et al. 2013). Though it is hard to estimate any timeframe, this suggests that future technology might be increasingly resilient to all sorts of disruptions that typically affect non-cooperative scenarios.

Non-cooperative scenarios can be considered with respect to the technical vulnerabilities involved, but they should also be analyzed with respect to their ethical and societal relevance. For instance, whether or not one should use such technology, if available, in what contexts, and to what extent, is object of intense discussion. In certain cases checking the reliability of testimonies through neurotechnology may be argued to be justifiably enforced for ‘the common good’ (Vedder and Klaming 2010). Contrary to the ‘for the common good’ reasoning, it has been argued that such scenarios could imply a violation of constitutional rights in certain legal systems (Pardo and Patterson 2013, ch. 6). For instance, they might constitute an infringement of the 4th and/or 5th amendment to the U.S. Constitution, that protect respectively against unreasonable searches and seizures and against self-incrimination.

## Conclusions

Brain reading technology represents a contemporary approach to mind reading. In principle, it grants the ability to read concealed mental states, possibly without a subject’s awareness or even cooperation. It would be important for a number of different stakeholders to be able to estimate the extent to which these possibilities are realistic, and the timeframe before they eventually become so. That is something which cannot be done once and for all. Rather, it takes consistent and competent monitoring of any and every relevant scientific and technological advancement. In order to be up to the task, ethicists, journalists and policy makers must be equipped with and agree upon reliable information and the appropriate conceptual tools. A meaningful and well-informed debate would contribute to prevent unnecessary concern among the public at large and allow the numerous interested stakeholders to timely and measuredly react to scientific and technological advances. It would also benefit neuroscientific research, as a more accurate understanding of the state of the art would contribute to maximize the confidence of institutions towards it.

Discussing the implications of a technology that is still in its infancy, runs the risk of being criticized as engaging in a premature discussion, or worse, even suggesting a technology is capable of more than current research warrants. Most of the ethically challenging scenarios that we envisage are currently only possible *in principle*, and ignore the technical limitations. Abstracting from current technology and technical limitation, according to this argument, might make our discussion less compelling and too speculative. Worse, it might fuel media hypes by either being too optimistic, creating unrealistic expectations, or, by being too pessimistic, generate unnecessary worries. We acknowledge such risks. At the same time, it would be unwise to wait with the assessment and discussion of potential implications of brain reading till the technology would be full-fledged. One shouldn’t delay the ethical discussion until it is too late (van de Poel and Royakkers 2011, p. 130). Societal debates take time too, and all too often technological (and economic) developments run ahead of proper societal evaluations to such an extent that it becomes extremely hard to correct them (consider e.g. the implications of internet tracking for privacy). Therefore, we suggest, one has no other option then to discuss the implications of technology under development, and it is important to do this as realistically as possible. It is for this reason that we suggest our evaluative criteria as ways of avoiding overly positive or excessively negative assessments of brain reading. Furthermore, our work might assist in identifying which research directions society would be (un)favorably disposed to.

We provided an overview of five aspects that we believe are among the most important ones to influence the practical applicability of a brain reading technology in practical scenarios, especially where mental privacy and civil rights are a concern. These aspects—accuracy, reliability, informativity, concealability and enforceability—could be used as criteria to produce an estimate of whether, when and to what situations brain reading technology could be applicable. Those criteria depend in turn on numerous ethical, legal and technical factors. Our discussion is aimed to identify several basic coordinates through which stakeholders can orient themselves within this rapidly growing field of brain reading. Numerous areas, such as healthcare, education and law, to name but a few, could benefit or suffer from brain reading technology’s novel possibilities. If this is the case for the present time, in the near future these possibilities are set to become even more compelling. We hope to promote general awareness of the basic concepts, criteria, methodology and applications of brain reading, and thereby facilitate a systematic discussion about its ethical, legal and societal implications.

## Footnotes

1 We will for now neglect the distinction between mental states and mental traits, and include both under the first term. We will return later in the text to make this distinction explicit.

2 These features were also discussed by Rorty (1970) when he considered whether they could be taken as special features (‘marks’) of the mental.

3 The words ‘method’ and ‘technology’ are not synonymous. Where a method is properly a comprehensive set of procedures or techniques and the connected theoretical knowledge, the word ‘technology’ puts a stronger accent to the material device that makes a certain task possible. However, we decided for simplicity’s sake to use the two terms interchangeably.

4 It is important to note that the concept of brain reading has been used in the literature with different degrees of generality. For instance, where Shen (2013) uses it in a more liberal fashion to include any form of brain measurement, Haynes (2012) tends to equal it to the concept of brain decoding. Since we aim at conceptual clarification, we will use ‘brain measurement’ for the most general version and ‘brain reading’ only for those applications aimed at decoding (classifying and interpreting) mental states.

5 BCI can also be said to entail implications for mental privacy (Ienca and Haselager 2016; Klein 2016). However, strictly speaking, working with BCIs in itself need not require one to interpret the brain signals in terms of mental states.

6 In brain reading, establishing the correctness of a certain result often implies subjective validation, i.e. asking the subject to confirm whether her mental state was really the one the system identified.

7 We will see in a later section why the subjects’ admission is not a detail.

8 The authors claim their method to be “somewhat” better, but the reported accuracy of phallometry varies across different studies.

9 In this case it is important to consider the different proneness to false positives and false negatives. A court might be inclined to risk by admitting a method that can lead to a small percentage of false negatives. On the contrary, it might be less eager to base a decision on or even only consider a method that is prone to any false positive, which could imply convicting an innocent person.

10 American Common Law has codified criteria for legal admissibility of scientific evidence in general. Those guidelines have been produced by the Supreme Court of the United States (Berger 2005) in cases like *Daubert v. Merrel Dow* (*Daubert v. Merrell Dow Pharmaceuticals (92*-*102), 509 U.S. 579* (1993)). The Supreme Court outlined four criteria to evaluate “scientific validity - and thus the evidentiary relevance and reliability” of a certain technology, theory or method: (a) whether it can be, and has been, tested, (b) whether or not it has been peer reviewed and published in scientific journals, (c) the error rate (and therefore the reliability) of the methods involved and (d) the degree of general acceptance within the relevant scientific community” (*Daubert v. Merrell Dow Pharmaceuticals (92-102), 509 U.S. 579* (1993)).

11 Similar concerns were valid already for classic techniques like polygraphic and psychological assessments. A subject, willing to deceive those methods, could learn how to control her physiological reactions (i.e. try to present a different signal), create confounds in the data by producing noise, or, in the case of questionnaires, to intentionally distort the outcome (Van Geert et al. 2016).

12 Daniel C. Dennett suggested such a scenario in a private conversation (Padua, May 2016).
